# African American Exposure to Prescribed Fire Smoke in Georgia, USA

**DOI:** 10.3390/ijerph16173079

**Published:** 2019-08-24

**Authors:** Cassandra Johnson Gaither, Sadia Afrin, Fernando Garcia-Menendez, M. Talat Odman, Ran Huang, Scott Goodrick, Alan Ricardo da Silva

**Affiliations:** 1 USDA Forest Service, Southern Research Station, Athens, GA 30602, USA; 2 Department of Civil, Construction, and Environmental Engineering, North Carolina State University, Raleigh, NC 27607, USA; 3 School of Civil and Environmental Engineering, Georgia Institute of Technology, Atlanta, GA 30332, USA; 4 Department of Statistics, University of Brasília, Brasília 70910-900, Brazil

**Keywords:** prescribed fire, environmental justice, socially vulnerable populations, Georgia

## Abstract

Our project examines the association between percent African American and smoke pollution in the form of prescribed burn-sourced, fine particulate matter (PM_2.5_) in the U.S. state of Georgia for 2018. (1) Background: African Americans constitute 32.4% of Georgia’s population, making it the largest racial/ethnic minority group in the state followed by Hispanic Americans at 9.8%. African Americans, Hispanic Americans, and lower wealth groups are more likely than most middle and upper income White Americans to be exposed to environmental pollutants. This is true because racial and ethnic minorities are more likely to live in urban areas where pollution is more concentrated. As a point of departure, we examine PM_2.5_ concentrations specific to prescribed fire smoke, which typically emanates from fires occurring in rural or peri-urban areas. Two objectives are specified: a) examine the association between percent African American and PM_2.5_ concentrations at the census tract level for Georgia, and b) identify emitters of PM_2.5_ concentrations that exceed National Ambient Air Quality Standards (NAAQS) for the 24-h average, i. e., >35 µg/m^3^. (2) Methods: For the first objective, we estimate a spatial Durbin error model (SDEM) where pollution concentration (PM_2.5_) estimates for 1683 census tracts are regressed on percent of the human population that is African American or Hispanic; lives in mobile homes; and is employed in agriculture and related occupations. Also included as controls are percent evergreen forest, percent mixed evergreen/deciduous forest, and variables denoting lagged explanatory and error variables, respectively. For the second objective, we merge parcel and prescribed burn permit data to identify landowners who conduct prescribed fires that produce smoke exceeding the NAAQS. (3) Results: Percent African American and mobile home dweller are positively related to PM_2.5_ concentrations; and government and non-industrial private landowners are the greatest contributors to exceedance levels (4) Conclusions: Reasons for higher PM_2.5_ concentrations in areas with higher African American and mobile home percent are not clear, although we suspect that neither group is a primary contributor to prescribed burn smoke but rather tend to live proximate to entities, both public and private, that are. Also, non-industrial private landowners who generated prescribed burn smoke exceeding NAAQS are wealthier than others, which suggests that African American and other environmental justice populations are less likely to contribute to exceedance levels in the state.

## 1. Introduction

Studies indicate that African Americans, Hispanic Americans, and lower socioeconomic status population groups in the U.S. are more likely than others either to live near polluting facilities or be exposed to greater amounts of ambient air pollutants [1,2]. In most cases, these studies focus on pollutants from urban or urban-proximate manufacturing facilities, given the concentration of racial and ethnic minority populations in urban centers [3,4,5]. Less research in the U.S. has emphasized human population exposure to pollution from land management activities such as prescribed burning that typically occur in rural contexts [6].

To address this gap in the literature, we use an environmental justice lens to examine the association between African Americans and prescribed burn particulate matter (PM_2.5_) concentrations with aerodynamic diameter of 2.5 μm or less in the U.S. state of Georgia. This exploratory analysis is intended to examine the fundamental relationship between African American presence and smoke exposure. A determination of the proportional impact of smoke on this population, which is consistent with environmental justice analyses, is beyond the scope of our analyses. Still, our query can be framed as an environmental critique because it provides a first look at African Americans’ spatial relationship to these activities in Georgia, which provides the basis for further investigations specifically examining disproportionate impacts. We also identify landowner types, i.e., government, conservation, non-industrial private landowners, and commercial entities, that produce PM_2.5_ concentrations that exceed National Ambient Air Quality Standards (NAAQS).

Objectives:

1. Specify a spatial regression model to examine the association between African Americans and prescribed fire-related PM_2.5_ concentrations in the U.S. state of Georgia
2. Identify dates, location, and emitters of prescribed fire-related PM_2.5_ concentrations that exceed the 24-h National Ambient Air Quality Standards for 1 January through 30 April 2018 in the U.S. state of Georgia

According to the 2018 National Prescribed Fire Use Activity Report, more than 1 million acres (~404,686 ha) of land were burned for forestry objectives alone in Georgia during 2017 [7]. More generally, prescribed fire activity in the southern U. S. (Georgia, Florida, Alabama, South Carolina) accounts for as much as 3–4 million hectares burned annually [8]. A prescribed burn (also called a controlled burn) is a planned fire conducted under specific conditions. One of the primary reasons prescribed fires are conducted is for hazardous fuels reduction, which minimizes the number and extent of larger, potentially catastrophic wildfires [9,10,11]. Florida and Georgia are the two eastern states in the U.S. South most impacted by wildland fire events contributing to premature deaths and respiratory-related hospital admissions [9]. A growing and aging population in these states, coupled with potential climate-induced increases in wildland fires [10], will necessitate effective smoke management practices like prescribed burning to help support healthy human populations [11].

In addition to fuel reductions, prescribed fire is used widely to enhance southern pine regeneration, improve wildlife habitat, control pests and associated disease dispersion, and to reduce invasive plant species [12]. With respect to pine regeneration, substantial efforts on the part of both public and private entities to restore the longleaf pine (*Pinus palustris*) and wiregrass ecosystem of Georgia’s coastal plain necessitate prescribed fire regimes [13,14,15]. Also, for much of the twentieth century, large south Georgia plantations have been host sites for wild game hunting (quail, deer, turkey), which again require periodic burning [16]. Certainly, a regional sub-culture has long been associated with plantation hunts and their overwhelmingly well-heeled participants [17].

Despite these benefits, prescribed fire also presents potential human health risks from PM_2.5_ concentrations [18]. By design, prescribed burn smoke lingers near the burn site rather than dispersing more rapidly like wildfires, which causes greater in situ human exposure. Atmospheric parameters such as mixing height, transport wind speed, and direction are critical in prescribed burn planning to select conditions that will transport smoke in a direction that minimizes human impact and rapidly disperses smoke [19]. The regularity of these burns also increases human exposure [18,20,21]. We are aware of only a few studies that have measured smoke distribution near or immediately downwind of prescribed burn sites [12,20,22,23]. Pearce et al. found that PM_2.5_ concentrations decreased significantly within 2 km of control burn sites but that in areas where smoke lingered, concentrations were 200% higher than their established baseline of 35 µg/m^3^. An Australian study found particulate matter concentrations exceeded air quality standards within 100 m of a smaller control burn (52 ha) and within 500 m of a larger fire (700 ha), suggesting that human exposure and resulting health impacts from prescribed burns can be significant [20,24]. Still, other studies question whether the physical and chemical composition of prescribed fires, because they are conducted under controlled conditions, present a relatively lower health hazard than wildfires [18,24].

Alongside the ecological and financial considerations of place that compel prescribed burning is a human dimension in the form of African American communities and cultures, also firmly embedded in rural Georgia [17,25]. African Americans constitute relatively large proportions of the population in some Georgia’s counties where these burning activities occur. For instance, a line of roughly thirty counties stretching from Wilkes County in the state’s northeast, Piedmont region, to Early County on the southwest border with Alabama, comprise most of Georgia’s “Black Belt”—defined as the “social and demographic crescent of southern geography containing a concentration of black people” with above average African American percentages [26] (p.2). In two of Georgia’s Black Belt counties, Dougherty and Baker, African Americans make up 70.2% and 45.5%, of the population, respectively, where prescribed burning is common on plantations and for agricultural purposes (Dougherty) and for longleaf pine restoration and agriculture (Baker) [27].

The state’s rural, African American communities are remnants of the South’s plantation-based economy and culture that utilized black farm laborers, first as enslaved people, who transitioned to sharecroppers/property owners and eventually to independent landowners during the latter portion of the 19th century and throughout the 20th century. At the height of African American rural landownership in 1910, blacks in Georgia were either full or part owners of 1,349,503 acres of farmland (546,124 ha), or roughly 5% of all farmland in the state [28]. Over the course of the 20th century and into the first decades of the 21st century, that number declined to 189,493 (76,685 ha) in 2017, representing 1.9% of all farmland in Georgia [29]. Farmland is less likely to be subjected to prescribed burning; however, the decline in agricultural ownership indicates the relative lack of black-owned rural land in the state that could be burned.

African Americans are not usually owners of large, rural estates and are mostly absent from the plantation–based hunting culture (except as caretakers), which suggests that, in South Georgia at least, African Americans are much less likely to participate in activities that contribute either directly or indirectly to smoke production from prescribed burning. Related studies from other southern states also indicate that low percentages of African Americans conduct prescribed burning [30,31].

## 2. Materials and Methods

### 2.1. Data Sources

*PM_2.5_ concentration*. We estimated daily, PM_2.5_ concentrations at 4 km spatial resolution associated with prescribed burning for 120 days spanning the first four months of the year, 1 January through 30 April 2018. This time period encompasses the majority of the state’s prescribed burning activity in any given year [32,33]. Our estimation method follows the data fusion approach developed by Friberg and colleagues [34] that combines: (1) observations at ambient monitors and (2) simulated pollutant concentrations from the Community Multiscale Air Quality model (CMAQ, v5.0.2, U.S. Environmental Protection Agency, Research Triangle Park, NC, USA) [35] at 4-km horizontal resolution. This technique produces fields that capture well spatiotemporal information from the air quality model, along with temporal variations from the pollutant observations. Model bias and errors are decreased as a result. The air quality impacts associated with prescribed burning were estimated using the Direct Decoupled Method (DDM) embedded in CMAQ [36], and the results of data fusion were scaled with the simulated prescribed fire impact to pollutant concentration ratios [37]. The resulting dependent variable, prescribed fire-derived PM_2.5_, is the four-month average concentration for 1693 census tracts.

*Prescribed fire burn permits and exceedance days.* The Georgia Institute of Technology and North Carolina State University created prescribed burn permit databases for Georgia and Florida for 2018 [37]. These data were assembled from records collected by the Georgia Forestry Commission (GFC) and the Florida Forest Service, respectively. According to the Air Protection Branch of the Georgia Environmental Protection Division (Georgia Department of Natural Resources), “[a]ll outdoor burning of natural vegetative materials is considered open burning and requires a burn permit from the Georgia Forestry Commission (GFC) “ [38]. The state allows for thirteen legal burn activities, including prescribed burns. Only permits for prescribed fires are included in our analyses.

Prescribed fire-related PM_2.5_ exceedance days were identified as those on which prescribed fire contributed to PM_2.5_ concentrations that exceeded 35 µg/m^3^ (24-hour average NAAQ for PM_2.5_) for at least one of the 4 km CMAQ grid cells covering Georgia—while the contribution of all other sources other than prescribed fire (non-fire) was lower than the NAAQS. To locate exceedance sources, we identified burn permits that occurred within 20 km from exceedance grid cells. We selected this distance to account for the effect of potential burn activity on exceedance at a given location from surrounding burns, while staying fairly close to a given exceedance site (cell) and considering that most burns are small intensity fires conducted under weather conditions that do not favor smoke dispersal [18,20].

*Landowner data.* To identify landowners contributing to PM_2.5_ exceedance, we combined CoreLogic and Digital Maps Products data for Georgia (years 2017–2018) to create a parcel layer that contained data on parcel owner, acreage (km), sale date, assessed value of improvements (e.g., buildings/homes), and assessed land value. These data were overlaid with locations of burn permits to identify landowners who likely contributed to PM_2.5_ exceedance. Landowners were classified into four broad categories--conservation, government, commercial, and non-industrial private landowners (NIPL). The conservation category includes national, state-specific, and local conservation groups, including plantations; government-owned land includes both federal and state property; commercial lands include industrial as well as small enterprises, and NIPL lands consist of residential landowners and non-industrial, smaller agricultural operations.

*Socio-demographic, industry, land cover data.* Explanatory variables include socio-demographic and industry data obtained from the 2011–2015 American Community at the census tract level. The socio-demographic variables are percent non-Hispanic African American, percent Hispanic, and percent living in manufactured housing; forest industry data is represented by percent of population 16 years or older employed in agriculture, forestry, fishing, hunting, and mining industries (AFFHM). Forest cover data are percent evergreen forest and percent mixed forest [39]. Forest cover data were obtained from the National Historic GIS (NHGIS) IPUMS land cover database [40].

### 2.2. Regression Analyses

We specify a spatial Durbin error regression model (SDEM) to analyze the association between percent African American and prescribed burn PM_2.5_, while controlling for percent Hispanic, manufactured housing, employment in natural resource-based industries, and forest cover. Manufactured housing is a socioeconomic status proxy, as mobile home dwellers are more likely to have lower income and education levels [41]. We also expect the greatest PM_2.5_ concentrations in areas where people are employed in land and forest-based industries like agriculture, forestry, fishing, hunting, and mining, and where there are more coniferous and mixed forests. Before the spatial modeling, we tested for spatial autocorrelation of residuals and explanatory variables in SAS 9.4, (SAS Institute Inc., Cary, NC USA) using linear regression models (ordinary least squares) and global Moran’s I as a measure of spatial clustering. Tests revealed a Moran’s I > 0 for all variables, indicating that model variables are not randomly distributed but rather cluster. Given such distributions, we determined that spatial regression models would provide the most robust parameter estimates.

We then tested four spatially explicit models [spatial Durbin, spatially lagged X, spatial autoregressive, spatial error] following LeSage and Pace [42,43], who recommend starting with a spatial Durbin model (SDM) of the form: y=ρWy+Xβ+WXΘ+ ε, where ρ is the coefficient for the spatially lagged vector of y_i_ values (i.e., the influence of neighboring PM_2.5_ concentrations on y_i_), and W is the associated distance-based weight; Xβ is a vector of explanatory variables in the area of interest; WXΘ is the influence of the neighboring vector of explanatory values on y_i_, with the associated distance-based weight of those influences, *W*. SDM imposes restrictions on model parameters, i.e., accounts for autocorrelation of the dependent and explanatory variables, respectively [44]. For the weight, we used an inverse distance of 10 km (Euclidian) to define the neighborhood (i.e., weight) for each observation. We used a specified distance weight because clusters of racial and ethnic groups, manufactured housing, and forest cover are based on distance rather than adjacency.

Model estimation proceeds by relaxing assumptions of autocorrelation for the y and x variables, i.e., ρ = 0 and Θ = 0. If ρ is restricted to 0, the SDM can be simplified to the spatially lagged X model (SLX): y=Xβ+WXΘ+ε, where only the explanatory variables are lagged; or, if Θ = 0, the SDM simplifies to the spatial auto regressive (SAR) model: y=ρWy+Xβ+ε, with only a spatially lagged dependent variable. The spatial error model (SEM) is derived by assuming Θ = −ρβ: y=Xβ+u, u=λWu+e and λ = ρ.

We also specified a spatial Durbin error (SDEM) as this model accounts simultaneously for auto-regression on both the explanatory and error terms, y=Xβ+WZΘ+ u, u=λW+ε; where Xβ is are vectors of explanatory variables and slopes, respectively, in the area of interest; ZΘ are slope vectors of neighboring explanatory variables; *W* is a weight matrix for the neighboring explanatory terms, and *u* accounts for autocorrelation of neighboring error terms with their associated weights [45]. See Table 1 for the equations and associated Akaike Information Criterion (AIC) values for each model [45].

We selected the SDEM because we were mostly interested in controlling for spatial clustering of the specified explanatory variables and error terms rather than the endogenous variable [46] (pp. 41–42). While we believe our explanatory variables are sufficient explicators of prescribed burn pollution, there are likely other, unobserved factors influencing or associated with prescribed burn PM_2.5_ emissions that we were not able to control for and operationalize. Importantly, these factors may vary in a non-random way, i.e., systematically across the state. We expect that both observed and unobserved factors in a given county influence prescribed burn PM_2.5_ emissions in that county and may also influence emissions in neighboring areas proximal to that space. Also affecting our selection is the real world consideration of prescribed fire smoke concentration and dispersion (dependent variable). As discussed, prescribed fire smoke tends to stay closer to the site of burn compared to wildfire smoke. We do not expect that PM_2.5_ emissions from a given census tract would affect PM_2.5_ concentrations in census tracts across the entire state of Georgia. Thus, a global lag Y model is not appropriate; rather, SDEM is the better model.

For spatial models, it is important to understand that interpretation of slope coefficients is not as straightforward as is the case for non-spatial models. LeSage [47] distinguishes between “local and global spillover” model specifications, with spillover referencing impacts from neighboring areas, however these are defined. Local, spatial spillover models include SLX, SEM, and SDEM with spatially lagged predictor variables, where there are both direct and indirect effects of the x variables on the dependent variable. The endogenous variable is impacted by x_1_, x_2_, x_3,_ etc. occurring in the same spatially-defined area as y_j_ (direct effect) and by spillover effects of x_1_, x_2_, x_3,_ in the larger, spatially defined neighborhood of y_j_ (indirect effect). In the case of a simply-defined, local model like SDEM, spillover or indirect effects are limited to y_i_; whereas global spillovers occur when a change in x_i_ in one area creates a ripple effect that causes changes to y_j_, not only in neighborhood A but changes in y_2,_ y_3,_ y_4_ etc. in neighborhoods throughout the entire study area (i.e., B, C, D, etc.) so that “a long-run steady state equilibrium arises” [47] (p.15). Both the direct and indirect slope coefficients for the SDEM model can be interpreted in a straightforward manner like linear model slopes [46] (p. 41).

## 3. Results

### 3.1. Linear and SDEM Models

Findings for objective 1 are in Table 2. The table contains results of both the Linear and SDEM models, with direct effects for the latter in column 1 and indirect effects in column 2. All predictors are highly significant in the linear model, and the amount of variance accounted for by the model is 36%. As expected, coefficients for employment and forest cover in both models are relatively higher than those for the social variables. We would expect that resource-based employment and forest cover would have higher coefficients, compared to other predictor variables. 

For SDEM, increases in percent African American and manufactured housing are associated with increased PM_2.5_ levels for that census tract only. The practical, direct effect of percent African American on PM_2.5_ output is assessed by examining black direct effects on the sample mean. For instance, mean PM_2.5_ concentration from 1 Jan to 30 April 2018 across all census tracts was 1.219 µg/m^3^. A 10% increase in the African American population for an average census tract would increase mean PM_2.5_ output in that tract by 0.04 µg/m^3^, an increase of about 3%; a 25% increase in black population would increase mean smoke pollution by 0.1 µg/m^3^, or roughly 8%. There is no significant indirect race effect, meaning that increasing African American percentages within 10 km of a given census tract would not increase pollution concentrations for the tract of interest. Another demographic variable, percent Hispanic, has a negative association with smoke pollution for both direct and indirect effects, but only the indirect effect is significant. Employment in extractive-based industries in both the referent and surrounding census tracts increases prescribed fire smoke, as does mixed forest cover; evergreen forests has only a direct impact. The significant lambda term indicates that neighboring error terms impact PM_2.5_ concentrations for the referent census tract. There are 1969 census tracts in Georgia, but the final model sample size was 1683 due to missing data for the various input datasets. 

The SDEM represents a relatively recent advancement in spatial modelling, as noted by Elhorst [48] (p.14), and even fewer applications have been applied to environmental justice analyses. However, in other topical areas, SDEM has been found to be a more robust model, compared to ordinary least squares, SLX, and SDM, for example in predicting voter turnout in the 2004 U.S. presidential election [49]. Also, in China, [50] Jiang et al. used an SDEM to look at energy efficiency in Chinese provinces [50], which like our model, indicated significant direct and indirect effects of predictors. As well, unobserved errors had a significant impact on energy efficiency. The link between percent African American and prescribed burning is consistent with Davies et al. [51], who found that wildfire vulnerability in areas of the country with moderate to high wildfire vulnerability increased significantly with increases in proportion African Americans, Hispanics, Native Americans, and other non-White racial groups. That modeling did not involve a spatial component.

### 3.2. Prescribed Fire PM_2.5_ Exceedance Days and Emitters

The following analyses address objective 2, the identification of prescribed fire PM_2.5_ emissions exceeding the 24-h NAAQS for 1 January through 30 April 2018 for Georgia. We found that for 34% of the days (41 out of 120), simulated PM_2.5_ concentration for at least one 4 km grid cell exceeded the daily standards with prescribed fire emissions contributing to exceedance. The number of grid cells with exceedance days are listed in Table 3. In addition, the average PM_2.5_ concentration, both total and prescribed fire-related, for these grid cells is included. On 10 March, the highest number of grid cells (631), covering an area over 10,000 km^2^, had prescribed fire-related PM_2.5_ exceedances. The *total* PM_2.5_ exceedance average includes both prescribed fire and non-prescribed fire PM_2.5_ emissions greater than 35 µg/m^3^; but for the *prescribed fire only* exceedance average, the mean includes days when prescribed fire PM_2.5_ was above 35 µg/m^3^ and days when non-prescribed fire PM_2.5_ concentrations were less than 35 µg/m^3^. For this reason, average prescribed fire PM_2.5_ on some days may not be higher than 35 µg/m^3^.

**Figure 1:**
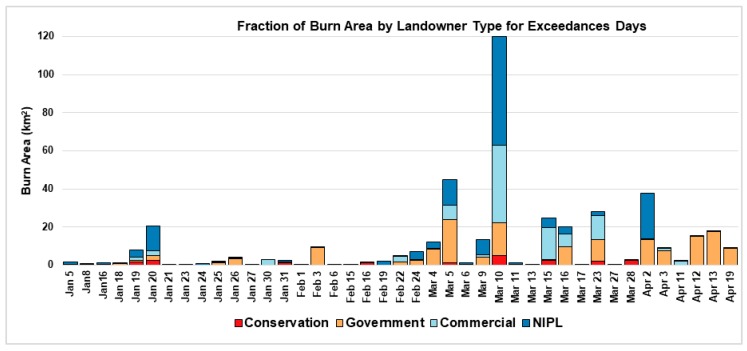
PM_2.5_ exceedance days in Georgia for 24-hour National Ambient Air Quality Standard (NAAQS) (1 January−30 April 2018), along with permitted burn area (Km^2^) categorized by landowner type. Burn area corresponds to the total burn area within a 20 km radius from the exceedance location, within 48 hours from the time of exceedance.

Figure 1 shows permitted burn area categorized by landowner type for each exceedance day. Burn area indicates total permitted burn area within 20 km from the exceedance grid cell(s) on that day and the day prior to exceedance. This figure shows the proportional contribution of the four landowning groups—conservation, government, commercial, and NIPL. The number of burn permits in each category were: 44 conservation, 145 government, 439 commercial, and 1199 NIPL. Conservation groups accounted for the smallest percentage of PM_2.5_ concentrations during exceedance days. In general, prescribed fires on NIPL, government, and commercial lands are most likely to cause an exceedance of the daily air quality standard. Again, 10 March 2018 was a day of exceptional activity for all landowning groups with the maximum number of fires (880 burn permits) occurring from 9 March to 10 March.

**Figure 2:**
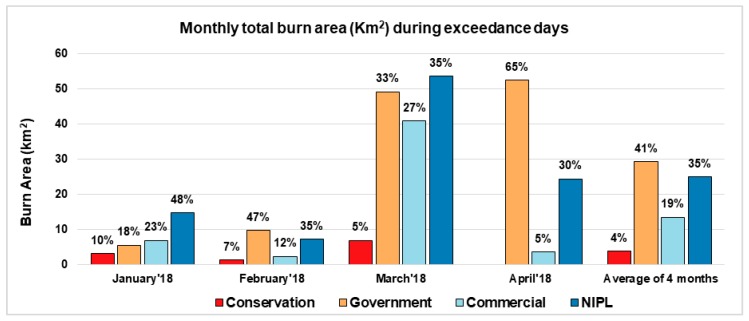
Monthly total burn area (km^2^) during the days of exceedance. For a specific month, the data label shows the relative percentage from the four landowner categories.

To distinguish the relative contribution by different types of landowners, we aggregated the permitted burn area for each exceedance day by month (Figure 2). The highest amount of area burned (above 50%) occurred during March, followed by April (28%). Similar to the daily pattern, fires on conservation land contributed less than 10% to the total burn area for all four months. Although the number of burn permits on government land is less than that of commercial lands, the four-month average burned area on government land is twice the area burned by commercial landowners. The amount of prescribed burn area accounted for by government landowners also increased steadily from January to April. This reflects the seasonality of prescribed burn activity.

**Figure 3:**
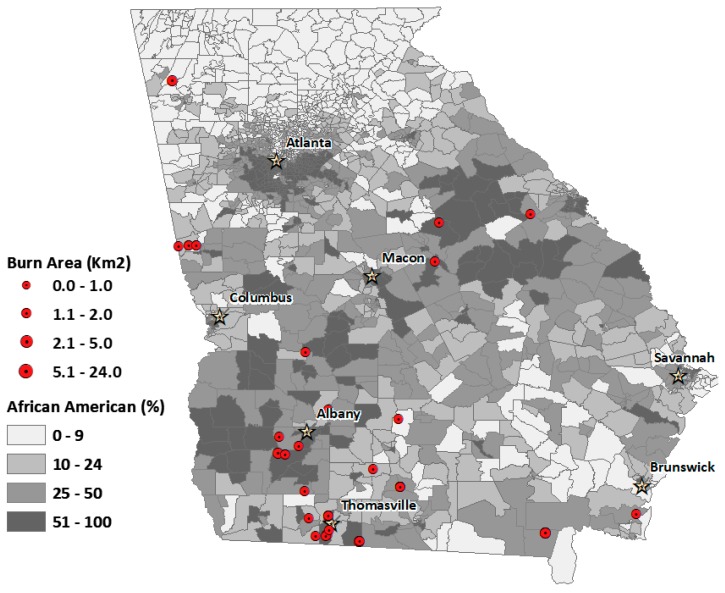
Spatial distribution of burn permits for conservation land exceedance days, overlaid with percent African American population for Georgia census tracts. Circles denote the relative burn area size for each permit.

**Figure 4:**
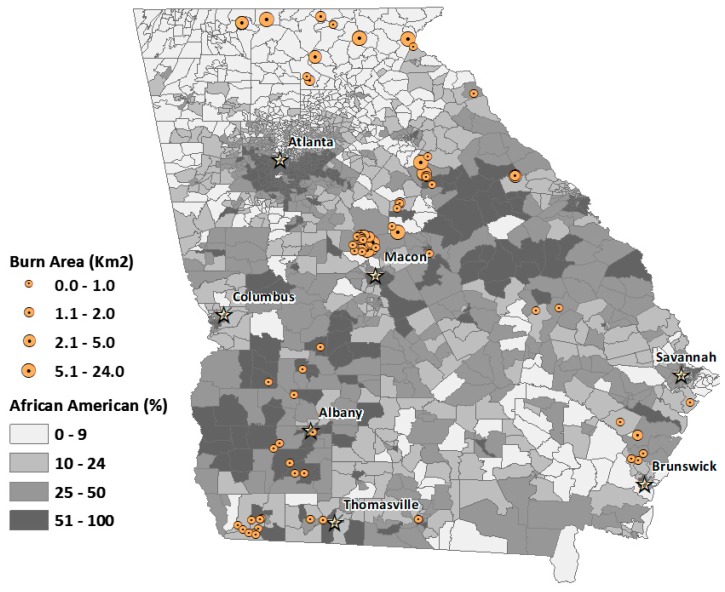
Spatial distribution of burn permits for government land exceedance days, overlaid with percent African American population for Georgia census tracts. Circles denote the relative burn area size for each permit.

**Figure 5:**
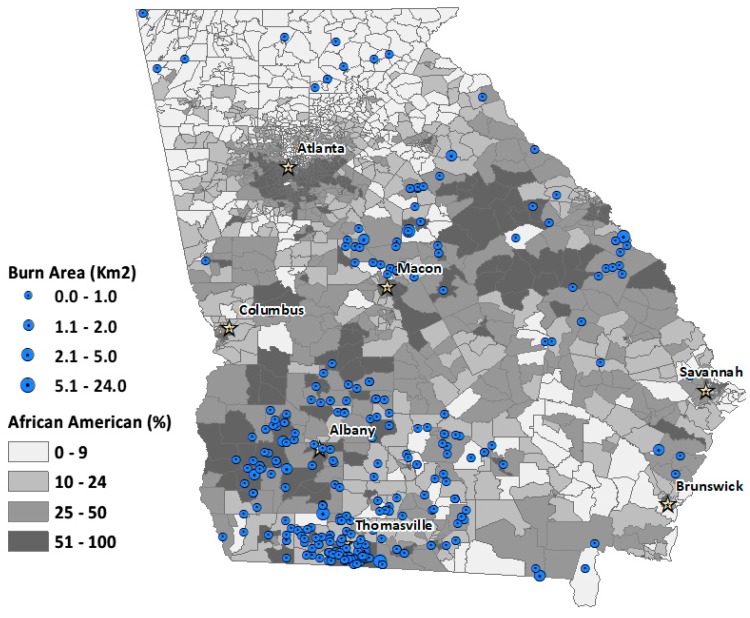
Spatial distribution of burn permits for commercial land exceedance days, overlaid with percent African American population for Georgia census tracts. Circles denote the relative burn area size for each permit.

**Figure 6:**
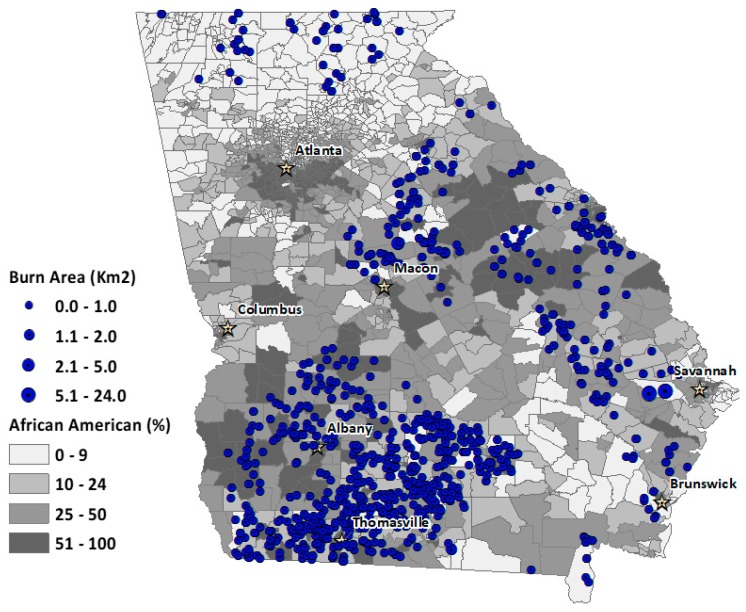
Spatial distribution of burn permits for non-industrial private landowner (NIPL) exceedance days, overlaid with percent African American population for Georgia census tracts. Circles denote the relative burn area size for each permit.

We also mapped the spatial distribution of permits contributing to degraded air quality over the four-month period for the four landowning classes (Figure 3, Figure 4, Figure 5 and Figure 6). The base map shows the percentage African American population at the census-tract level. This context of racial distribution is provided to show prescribed fire exceedance relative to the state’s African American population. While some metropolitan Atlanta census tracts have very high African American percentages, higher African American percentages also occur in census tracts corresponding to the state’s more rural Black Belt areas, again, stretching from the Piedmont in the northeast to southwest Georgia. The circles indicate the location of burn permits within 20 km from the location of an exceedance grid for a specific landowning class. The varying circle sizes reflect the size of area burned for a given permit. 

Generally, permits associated with air quality exceedance are more numerous in tracts with higher percentages of rural African American populations, particularly for commercial and NIPL permits (See Figure 5 and Figure 6).

Of the 34 governmental agencies identified, 32% (11 out of 34) were federal, and 68% (23 out of 34) were non-federal, but land area burned by the respective governments was: 1674 km^2^ (federal), compared to 759 km^2^ (non-federal). The largest government burns appear to be in north Georgia (associated with burning on national forests) and in middle Georgia. The landowner data indicates that the cluster of burn permits in middle Georgia, just to the north of Macon, are titled “United States Government.” These permits are likely for the U.S. Fish and Wildlife Service’s, Piedmont National Wildlife Refuge. The Refuge conducts extensive burning to support red-cockaded woodpecker populations.

NIPL data (Figure 6) do not contain racial identifiers, so we were not able to determine the racial representation of exceedance emitters; however, mean, assessed land value for NIPL exceeders was $166,526 and mean acres, 205 (82.96 ha). This compares to a mean, assessed land value of $154,112 and mean acres of 148 (59 ha) for NIPLs generally in the state. These differences indicate that, on average, NIPL exceeders were wealthier than other NIPLs in the state.

## 4. Discussion

This is the first study of which we are aware that examines African American exposure to PM_2.5_ concentrations from prescribed burns. As such, it opens a dialogue about the impact of natural resource management activities on environmental justice populations. In terms of the first objective, spatial regression results indicate that African American presence is positively associated with PM_2.5_ concentrations, as is mobile home residence. Why might this be the case—are these population groups more likely to conduct prescribed burns? In terms of African American burning, again prior research would suggest that blacks do not typically burn. Also, the first author conducted three informal interviews with southwest Georgia African American agricultural landowners, who relayed that local African Americans often lease their farms (which in some cases include woodlands) to White landowners to cultivate. They do so because of long-held notions that these arrangements are more profitable than if they farmed themselves. The farmers interviewed disagreed with this presumption, given assistance from various federal agencies to assist smaller farmers; however, the interviewed farmers stressed that it was difficult to convince many of their neighbors that higher profits could be had by farming. We suspect that in rural parts of the state with higher African American populations, larger White landowners are conducting burns on their own land and burning land leased from blacks. We also suspect that the positive association for both African Americans and mobile home dwellers is due to the co-location of these populations with rural, government-owned and conservation land where burning is routine.

We also identified landowners associated with elevated PM_2.5_ emissions. The aim here was to obtain a better understanding of the broad category of prescribed burn PM_2.5_ emitters who may be more likely to degrade air quality. Again, NIPLs exceeding air quality standards had higher average land values and acreage than NIPLs generally. In terms of environmental justice implications, these findings would lead one to conclude that lower wealth landowners are less likely to be numbered among exceedance NIPLs because the former, by definition, have lower valued land assets. For similar reasons, African American and other minority landowners are less likely to be in the exceedance category. In the government category, the federal government was the primary exceeder, and findings indicate that the federal government is not a non-trivial contributor to elevated PM_2.5_ emissions from prescribed burning in Georgia.

## 5. Conclusions

Federal guidelines instruct that prescribed smoke impacts be minimized to “smoke sensitive” facilities such as schools, hospitals, nursing homes, airports, or heavily populated areas [52]. In addition, 1994 Executive Order 12,898 and its amendments direct federal agencies to identify and address “disproportionately high and adverse human health or environmental effects of [their] programs, policies, and activities on minority populations and low-income populations….” [53]. As indicated, prescribed burns are conducted according to specific guidelines that consider meteorological and other factors. These factors equal, burns conducted by public agencies sometimes involve subjective decisions [6] that may result in smoke exposure to communities that voice fewer complaints about smoke or that have less political and social influence. Rural minority communities typically have less persuasion in such situations [54]. Our study did not assess whether African Americans were disproportionately exposed to prescribed burn smoke, but findings beg the question of whether these burns are conducted at some cost to nearby human populations, in particular those who are less likely to generate burns or to benefit economically or socially from the same.

Exceedance levels notwithstanding, if considered from the viewpoint of wildfire mitigation, an argument could be made that prescribed burns actually enhance environmental justice for nearby populations. As discussed, the chances of larger, more severe wildfire is lessened on properties that are burned regularly, and this can of benefit to environmental justice populations [6]. We certainly do not dispute that wildfire risk is reduced by prescribed burn applications; however, framing prescribed burning *only* in terms of an environmental benefit does not address the possible smoke hazards from prescribed fire. A pertinent question then is: does wildfire risk reduction via prescribed burning outweigh the imposition of cumulative smoke exposure for affected populations? We recommend that future research examine whether environmental justice populations, more broadly, are disproportionately exposed to prescribed fire smoke and whether such exposure exceeds national air quality standards.

## Figures and Tables

**Table 1:** Spatial Regression Models and Tests for Optimal Model Fit.

Model Name	Equations	Akaike Information Criterion
Linear	y=Xβ+ε	2120
SDM	y=ρWy+Xβ+WXΘ+ε	956
SLX	y=XΒ+WXΘ+ε	1978
SAR	y=ρWy+Xβ+ε	1476
SEM	y=Xβ+u, u=λWu+ε	661
SDEM	y=Xβ+WZΘ+u, u=λW+ε	626

**Table 2:** Linear Model and Spatial Durbin Error Model. *n* = 1683.

Variable	Linear Model	Spatial Durban Error Model	
	Beta	Direct Effect	Indirect Effect
% black	0.01 ****	0.004 ****	−0.001
	(0.0004)	(0.0004)	(0.001)
% Hispanic	−0.01 ****	−0.001	−0.010 **
	(0.001)	(0.001)	(0.002)
% mobile home	0.01 ****	0.003 ****	0.004
	(0.001)	(0.001)	(0.002)
% AFFHM	0.05 ****	0.037 ****	0.065 ****
	(0.004)	(0.003)	(0.007)
% evergreen	1.02 ****	0.790 ****	−0.396
	(0.147)	(0.113)	(0.282)
% mixed	2.72 ****	1.157 ****	3.073 **
	(0.385)	(0.343)	(0.924)
λ	--	--	0.827 ****
	--	--	(0.014)
R^2^	0.36	--	--

Number in parenthesis is standard error. **** *p* ≤ 0.0001; ** *p* ≤ 0.01.

**Table 3:** Exceedance days along with the number of grid cells and the average particulate matter (PM2.5) concentration.

Exceedance Day	Area of Grid Cells with Exceedance (Km^2^)	Average Total PM_2.5_ in Exceedance Grid Cells	Average Prescribed Fire PM_2.5_ in Exceedance Grid Cells	Exceedance Day	Area of Grid Cells with Exceedance (Km^2^)	Average Total PM_2.5_ in the Exceedance Grid Cells	Average Prescribed Fire PM_2.5_ in Exceedance Grid Cells
**5-Jan-18**	32	58	55	**4-Mar-18**	304	68	65
**8-Jan-18**	48	54	53	**5-Mar-18**	1568	41	30
**16-Jan-18**	16	38	34	**6-Mar-18**	16	35	1
**18-Jan-18**	208	70	65	**9-Mar-18**	48	43	40
**19-Jan-18**	752	66	58	**10-Mar-18**	10,096	39	19
**20-Jan-18**	1584	40	31	**11-Mar-18**	32	36	4
**21-Jan-18**	32	42	23	**13-Mar-18**	48	58	53
**23-Jan-18**	48	75	74	**15-Mar-18**	64	38	18
**24-Jan-18**	16	44	42	**16-Mar-18**	240	45	38
**25-Jan-18**	16	37	34	**17-Mar-18**	80	40	11
**26-Jan-18**	16	37	31	**23-Mar-18**	80	52	48
**27-Jan-18**	16	36	34	**27-Mar-18**	16	45	44
**30-Jan-18**	224	68	65	**28-Mar-18**	16	48	45
**31-Jan-18**	32	42	37	**2-Apr-18**	272	70	68
**1-Feb-18**	16	37	34	**3-Apr-18**	16	41	39
**3-Feb-18**	48	52	50	**11-Apr-18**	896	108	105
**6-Feb-18**	16	35	7	**12-Apr-18**	96	53	49
**15-Feb-18**	32	65	61	**13-Apr-18**	32	42	40
**16-Feb-18**	16	39	38	**19-Apr-18**	16	49	48
**19-Feb-18**	16	51	49				
**22-Feb-18**	176	65	64				
**24-Feb-18**	16	45	43				
